# Supplementary material for: Comprehensive analysis of central carbon metabolism illuminates connections between nutrient availability, growth rate, and cell morphology in Escherichia coli
Source: PLoS Genet. 2018 Feb 12;14(2):e1007205. doi: 10.1371/journal.pgen.1007205 (PMC5825171; doi:10.1371/journal.pgen.1007205)
Supplement: S1 Table — (DOCX) [file pgen.1007205.s001.docx]

**Table S1.** Bacterial strains used in this study

| Annotation | Relevant genotype | Source | Keio Source ID [1] |
| --- | --- | --- | --- |
| MG1655 | *F^-^ λ^-^ ilvG- rfb-50 rph-1* | [2] |  |
| BH330 | MG1655 *P_lac_*::*gfp-ftsZ* (*bla*) | [3] |  |
| PAL2452 | MG1655, *leu*82::*Tn10* *ftsZ84*(ts) | [4] |  |
| EC440 | MC4100 Δ(λ*attL*-*lom*)::*bla* *lacI*q P*_lac_*::*gfp*-*ftsN* | [5] |  |
| EAM621 | MG1655 Δ(λ*attL*-*lom*)::*bla* P*_lac_*::*gfp*-*ftsN* | P1(EC440) x MG1655 |  |
|  |  |  |  |
| CSW15 | MG1655 *sucC*::*kan* | P1(JW0717) x MG1655 | JW0717 |
| CSW21 | MG1655 *aceE*::*kan* | P1(JW0110) x MG1655 | JW0110 |
| CSW26 | MG1655 *tpiA*::*kan* | P1(JW3890) x MG1655 | JW3890 |
| CSW36 | MG1655 *gpmM*::*kan* | P1(JW3587) x MG1655 | JW3587 |
| CSW52 | MG1655 *pfkA*::*kan* | P1(JW3887) x MG1655 | JW3887 |
| CSW56 | MG1655 *pgi*::*kan* | P1(JW3985) x MG1655 | JW3985 |
| CSW87 | MG1655 *icd*::*kan* | P1(JW1122) x MG1655 | JW1122 |
| CSW89 | MG1655 *tktA*::*kan* | P1(JW5478) x MG1655 | JW5478 |
| CSW91 | MG1655 *rpiB*::*kan* | P1(JW4051) x MG1655 | JW4051 |
| CSW93 | MG1655 *talB*::*kan* | P1(JW0007) x MG1655 | JW0007 |
| CSW95 | MG1655 *glpX*::*kan* | P1(JW3896) x MG1655 | JW3896 |
| CSW97 | MG1655 *pykA*::*kan* | P1(JW1843) x MG1655 | JW1843 |
| CSW99 | MG1655 *pgl*::*kan* | P1(JW0750) x MG1655 | JW0750 |
| CSW101 | MG1655 *edd*::*kan* | P1(JW1840) x MG1655 | JW1840 |
| CSW103 | MG1655 *gltA*::*kan* | P1(JW0710) x MG1655 | JW0710 |
| CSW105 | MG1655 *pfkB*::*kan* | P1(JW5280) x MG1655 | JW5280 |
| CSW108 | MG1655 *fbp*::*kan* | P1(JW4191) x MG1655 | JW4191 |
| CSW110 | MG1655 *rpiA*::*kan* | P1(JW5475) x MG1655 | JW5475 |
| CSW112 | MG1655 *ybhA*::*kan* | P1(JW0749) x MG1655 | JW0749 |
| CSW114 | MG1655 *fbaB*::*kan* | P1(JW5344) x MG1655 | JW5344 |
| CSW116 | MG1655 *eda*::*kan* | P1(JW1839) x MG1655 | JW1839 |
| CSW118 | MG1655 *tktB*::*kan* | P1(JW2449) x MG1655 | JW2449 |
| CSW120 | MG1655 *yggF*::*kan* | P1(JW2897) x MG1655 | JW2897 |
| CSW124 | MG1655 *talA*::*kan* | P1(JW2448) x MG1655 | JW2448 |
| CSW130 | MG1655 *sdhA*::*kan* | P1(JW0713) x MG1655 | JW0713 |
| CSW136 | MG1655 *fumB*::*kan* | P1(JW4083) x MG1655 | JW4083 |
| CSW138 | MG1655 *mqo*::*kan* | P1(JW2198) x MG1655 | JW2198 |
| CSW148 | MG1655 *mdh*::*kan* | P1(JW3205) x MG1655 | JW3205 |
| CSW150 | MG1655 *aceB*::*kan* | P1(JW3974) x MG1655 | JW3974 |
| CSW152 | MG1655 *sucA*::*kan* | P1(JW0715) x MG1655 | JW0715 |
| CSW156 | MG1655 *fumA*::*kan* | P1(JW1604) x MG1655 | JW1604 |
| CSW160 | MG1655 *gnd*::*kan* | P1(JW2011) x MG1655 | JW2011 |
| CSW166 | MG1655 *pykF*::*kan* | P1(JW1666) x MG1655 | JW1666 |
| CSW176 | MG1655 *cyaA*::*kan* | P1(JW3778) x MG1655 | JW3778 |
| CSW178 | MG1655 *crp*::*kan* | P1(JW5702) x MG1655 | JW5702 |
| CSW194 | MG1655 *zwf*::*kan* | P1(JW1841) x MG1655 | JW1841 |
| CSW196 | MG1655 *pta*::*kan* | P1(JW2294) x MG1655 | JW2294 |
| CSW198 | MG1655 *acs*::*kan* | P1(JW4030) x MG1655 | JW4030 |
| CSW200 | MG1655 *eutD*::*kan* | P1(JW2442) x MG1655 | JW2442 |
| CSW202 | MG1655 *ackA*::*kan* | P1(JW2293) x MG1655 | JW2293 |
| PAL3332 | MG1655 *crr*::*kan* | P1(JW2410) x MG1655 | JW2410 |
| PAL3592 | MG1655 *glk*::*kan* | P1(JW2385) x MG1655 | JW2385 |
| PAL3598 | MG1655 *acnA*::*kan* | P1(JW1268) x MG1655 | JW1268 |
| PAL3600 | MG1655 *acnB*::*kan* | P1(JW0114) x MG1655 | JW0114 |
| PAL3602 | MG1655 *rpe*::*kan* | P1(JW3349) x MG1655 | JW3349 |
| PAL4042 | MG1655 *bolA*::*kan* | P1(JW5060) x MG1655 | JW5060 |
| EAM114 | MG1655 *ompR*::*kan* | P1(JW3368) x MG1655 | JW3368 |
|  |  |  |  |
| CSW321 | PAL2452 *sucC*::*kan* | P1(JW0717) x PAL2452 |  |
| CSW694 | PAL2452 *crr*::*kan* | P1(JW2410) x PAL2452 |  |
| CSW700 | PAL2452 *aceE*::*kan* | P1(JW0110) x PAL2452 |  |
| CSW331 | PAL2452 *tktA*::*kan* | P1(JW5478) x PAL2452 |  |
| CSW335 | PAL2452 *pgl*::*kan* | P1(JW0750) x PAL2452 |  |
| CSW333 | PAL2452 *rpiA*::*kan* | P1(JW5475) x PAL2452 |  |
| CSW325 | PAL2452 *rpe*::*kan* | P1(JW3349) x PAL2452 |  |
| CSW327 | PAL2452 *pta*::*kan* | P1(JW2294) x PAL2452 |  |
| CSW698 | PAL2452 *ackA*::*kan* | P1(JW2293) x PAL2452 |  |
| CSW363 | PAL2452 *acnB*::*kan* | P1(JW0114) x PAL2452 |  |
| CSW339 | PAL2452 *zwf*::*kan* | P1(JW1841) x PAL2452 |  |
| CSW315 | PAL2452 *yggF*::*kan* | P1(JW2897) x PAL2452 |  |
| CSW925 | PAL2452 *talB*::*kan* | P1(JW0007) x PAL2452 |  |
| CSW926 | PAL2452 *eda*::*kan* | P1(JW1839) x PAL2452 |  |
| CSW731 | PAL2452 *crp*::*kan* | P1(JW5702) x PAL2452 |  |
| CSW733 | PAL2452 *cyaA*::*kan* | P1(JW3778) x PAL2452 |  |
| CSW923 | PAL2452 *gnd*::*kan* | P1(JW2011) x PAL2452 |  |
|  |  |  |  |
| CSW913 | BH330 *sucC*::*kan* | P1(JW0717) x BH330 |  |
| CSW909 | BH330 *crr*::*kan* | P1(JW2410) x BH330 |  |
| CSW229 | BH330 *aceE*::*kan* | P1(JW0110) x BH330 |  |
| CSW223 | BH330 *tktA*::*kan* | P1(JW5478) x BH330 |  |
| CSW227 | BH330 *pgl*::*kan* | P1(JW0750) x BH330 |  |
| CSW919 | BH330 *rpiA*::*kan* | P1(JW5475) x BH330 |  |
| CSW921 | BH330 *rpe*::*kan* | P1(JW3349) x BH330 |  |
| CSW215 | BH330 *pta*::*kan* | P1(JW2294) x BH330 |  |
| CSW231 | BH330 *ackA*::*kan* | P1(JW2293) x BH330 |  |
| CSW219 | BH330 *acnB*::*kan* | P1(JW0114) x BH330 |  |
| CSW217 | BH330 *zwf*::*kan* | P1(JW1841) x BH330 |  |
| CSW225 | BH330 *yggF*::*kan* | P1(JW2897) x BH330 |  |
| CSW905 | BH330 *talB*::*kan* | P1(JW0007) x BH330 |  |
| CSW907 | BH330 *eda*::*kan* | P1(JW1839) x BH330 |  |
| CSW233 | BH330 *tpiA*::*kan* | P1(JW3890) x BH330 |  |
| CSW846 | BH330 *gpmM*::*kan* | P1(JW3587) x BH330 |  |
| CSW917 | BH330 *crp*::*kan* | P1(JW5702) x BH330 |  |
| CSW915 | BH330 *cyaA*::*kan* | P1(JW3778) x BH330 |  |
| CSW911 | BH330 *gnd*::*kan* | P1(JW2011) x BH330 |  |
|  |  |  |  |
| CSW943 | EAM621 *aceE*::*kan* | P1(JW0110) x EAM621 |  |
| CSW944 | EAM621 *ackA*::*kan* | P1(JW2293) x EAM621 |  |
| CSW945 | EAM621 *pta*::*kan* | P1(JW2294) x EAM621 |  |

P1 transduction is described as: P1(donor strain) x recipient strain

**Table S1 References**

1. Baba T, Ara T, Hasegawa M, Takai Y, Okumura Y, et al. Construction of *Escherichia coli* K-12 in-frame, single-gene knockout mutants: the Keio collection. Mol Syst Biol. EMBO Press; 2006;2: 2006.0008. doi:10.1038/msb4100050.

2. Guyer MS, Reed RR, Steitz JA, Low KB. Identification of a sex-factor-affinity site in *E. coli* as gamma delta. Cold Spring Harb Symp Quant Biol. 1981;45 Pt 1: 135-140.

3. Hill NS, Buske, PJ, Shi Y, Levin PA A Moonlighting Enzyme Links Escherichia coli Cell Size with Central Metabolism. PLoS Genet. Public Library of Science; 2013;9: e1003663. doi:10.1371/journal.pgen.1003663.

4. Richard M, Hirota Y. Process of cellular division in Escherichia coli: physiological study on thermosensitive mutants defective in cell division. J Bacteriol. 1973;116: 314-322.

5. Gerding MA, Liu B, Bendezu FO, Hale CA, Bernhardt TA, de Boer PAJ. Self-enhanced accumulation of FtsN at division sites and roles for other proteins with a SPOR domain (DamX, DedD, and RlpA) in Escherichia coli cell constriction. J Bacteriol. 2009;191: 7383-7401. doi:10.1128/JB.00811-09.
