# Supplementary figures and images for: Comprehensive analysis of central carbon metabolism illuminates connections between nutrient availability, growth rate, and cell morphology in Escherichia coli
Source: PLoS Genet. 2018 Feb 12;14(2):e1007205. doi: 10.1371/journal.pgen.1007205 (PMC5825171; doi:10.1371/journal.pgen.1007205)

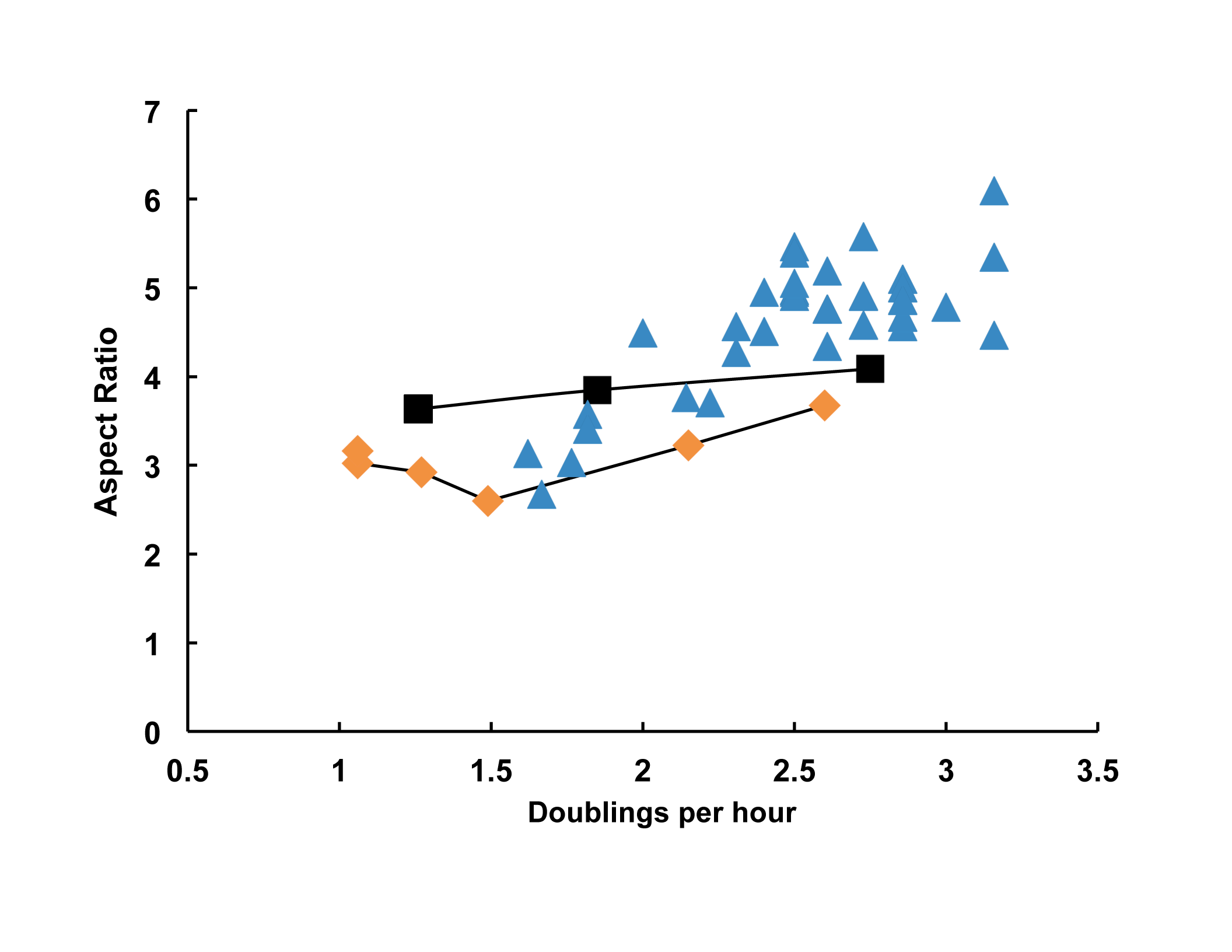

Supplement: S1 Fig — Length and width data from Table 1 were used to generate the aspect ratio for each CCM mutant (blue triangles). Aspect ratio data for different media was generated from S3 Table (black squares) or from previously reported results (orange diamond) [6]. (TIF) [file pgen.1007205.s005.tif]

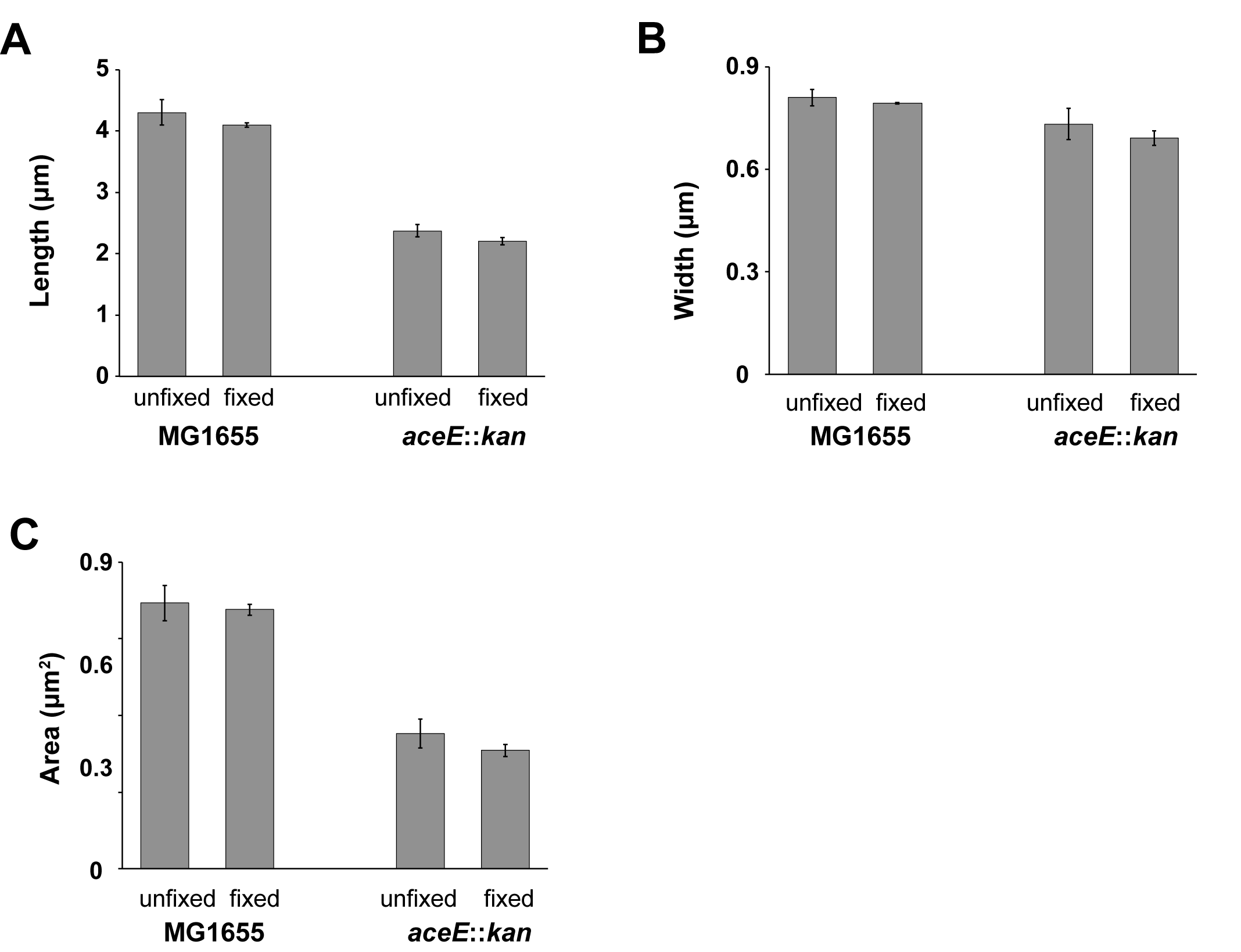

Supplement: S2 Fig — MG1655 and aceE::kan cells were grown in LB-glu and were either directly measured (unfixed) or fixed with paraformaldehyde-glutaraldehyde then measured. Average cell length (A), width (B), or area (C) are shown. Data is from three independent experiments with >200 cells measured per experiment. Error bars represent the standard error of the mean. (TIF) [file pgen.1007205.s006.tif]

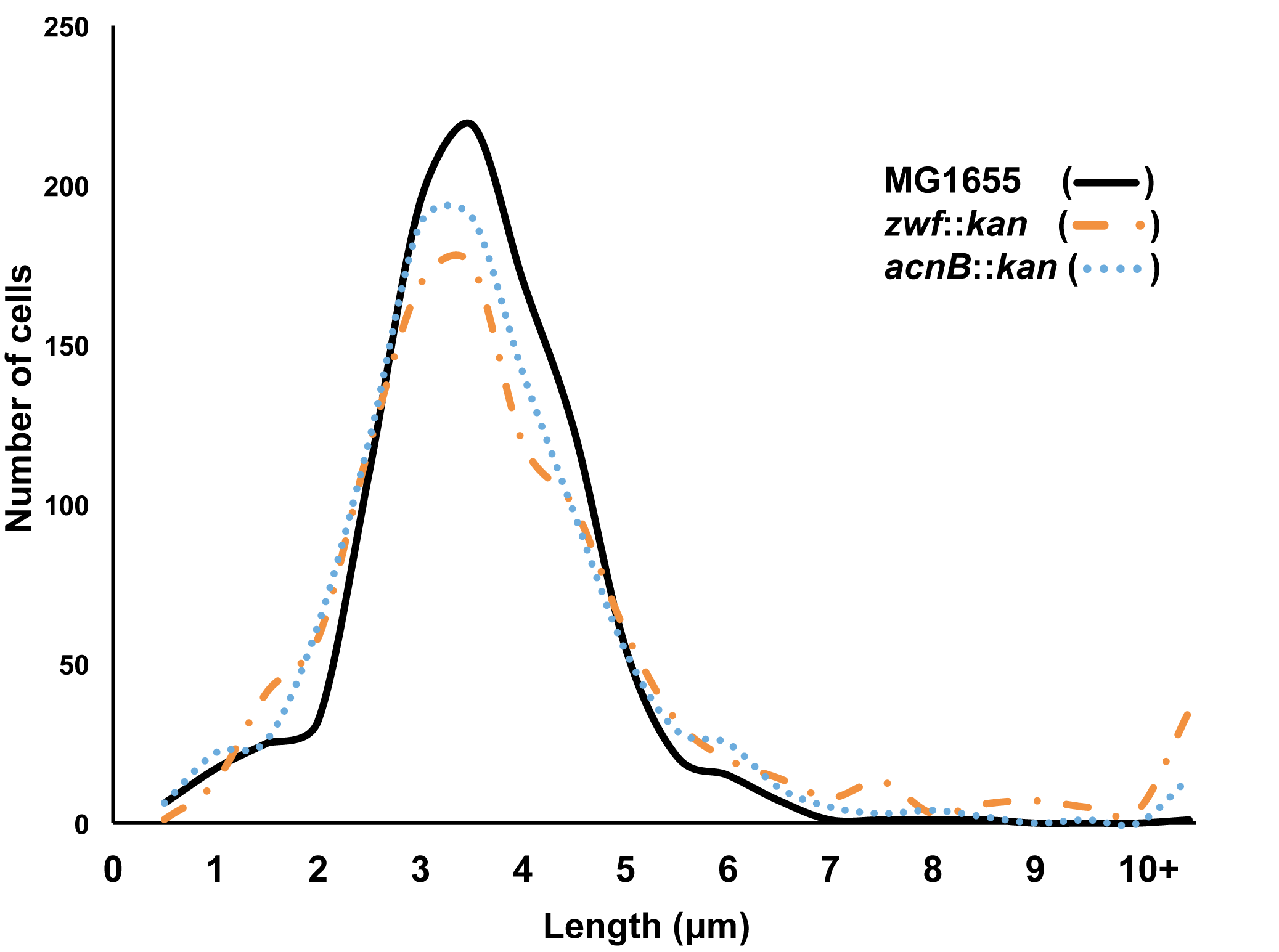

Supplement: S3 Fig — Length data of 1000 cells from the LB-glu growth condition from S3 Table was binned into 0.5 μm bins for MG1655 (black line), zwf::kan (orange dash-dot) and acnB::kan (blue dots). (TIF) [file pgen.1007205.s007.tif]

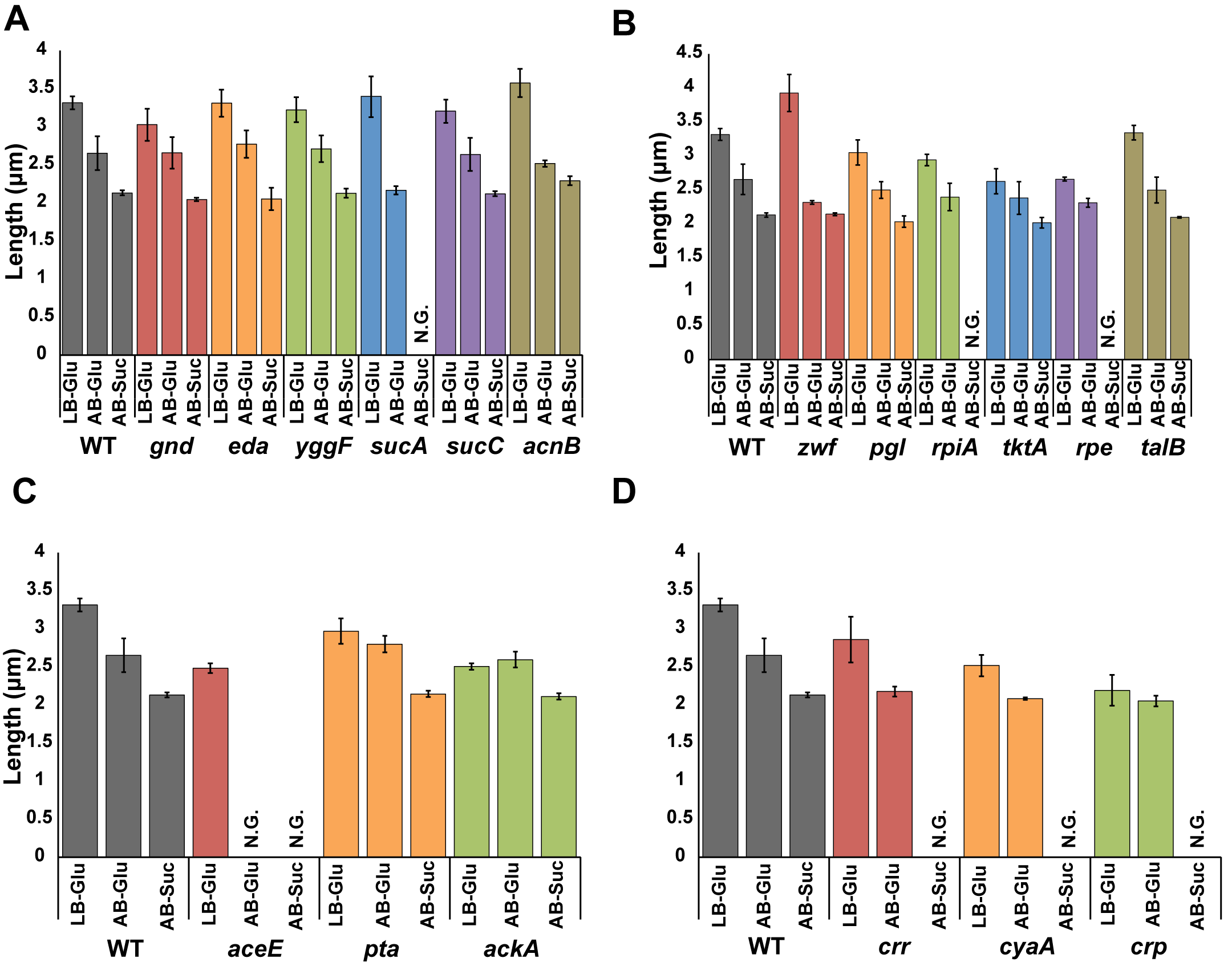

Supplement: S4 Fig — Average length data for the CCM mutants grown in LB-glu, AB-glu, and AB-suc divided into Entner-Doudoroff, gluconeogenesis, and TCA (A), pentose phosphate pathway (B), acetate fermentation (C), cAMP synthesis (D). Error bars represent standard error of the mean. Data is from three independent experiments with >200 cells measured per experiment. N.G. indicates no growth in the condition shown. (TIF) [file pgen.1007205.s008.tif]

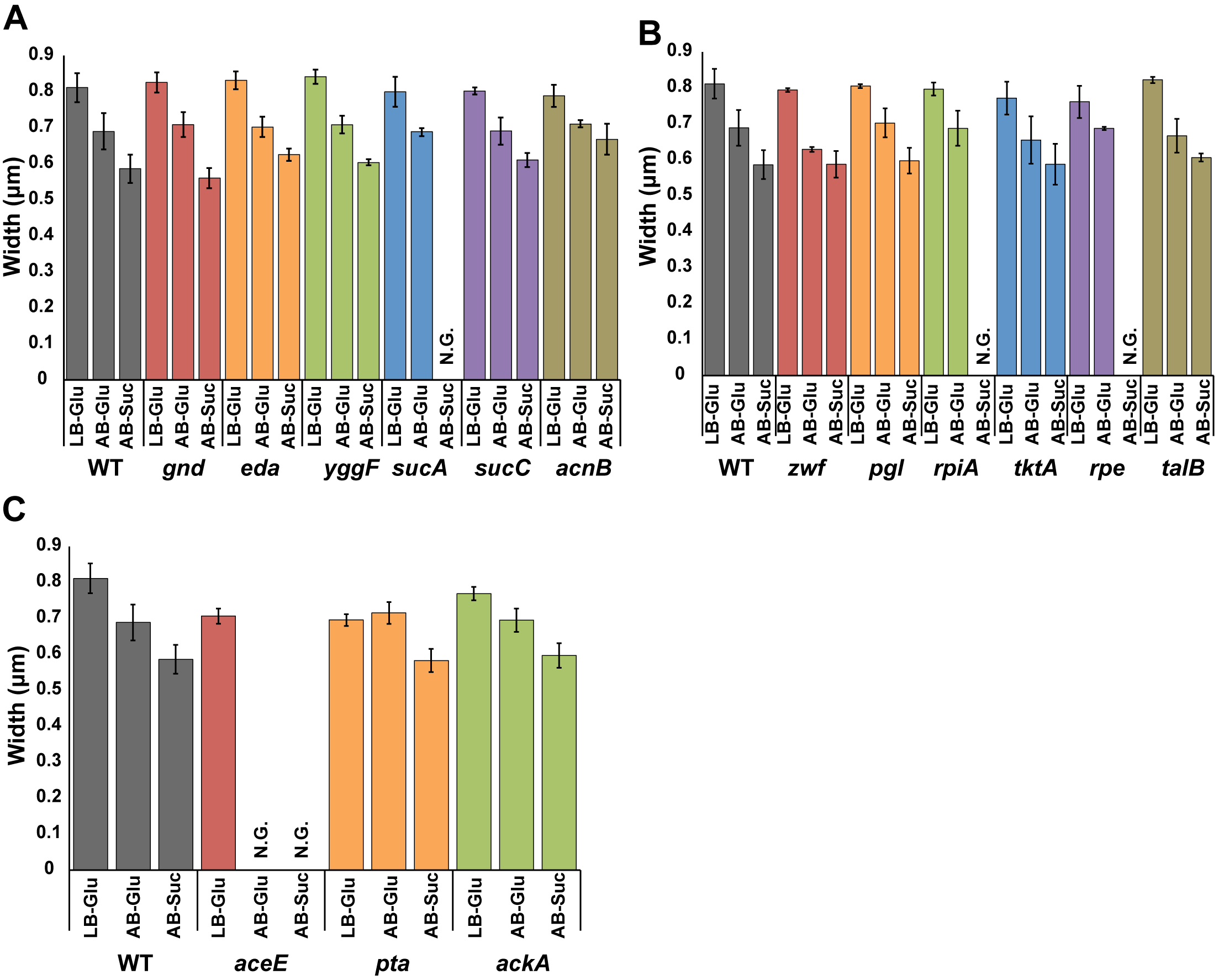

Supplement: S5 Fig — Average width data for the CCM mutants in LB-glu, AB-glu, and AB-suc divided into Entner-Doudoroff, gluconeogenesis, and TCA (A), pentose phosphate pathway (B), and acetate fermentation (C). cAMP mutants are shown in Fig 3E. Wild-type MG1655 data is shown in each panel to aid in comparison. Error bars represent standard error of the mean. Data is from three independent experiments with >200 cells measured per experiment. N.G. indicates no growth in condition shown. (TIF) [file pgen.1007205.s009.tif]
